# Everybody's Page

**Published:** 1889-01-05

**Authors:** 


### Everybody's Page.

People who find soap irritating to the skin should use a teaspoonful of sal volatile or a few drops of spirit of ammonia in a quart of distilled water.

A method of curing drunkenness, which has frequently been successful, is to put a small dose of some emetic in everything the inebriate drinks till the frequent sickness causes him to feel dislike and terror at the very sight of intoxicants.

Some people are almost too sensitive to the fitness of things. An old woman went to buy a pound of tea. "Will you have black tea, or green tea?" asked the shopman "Well," she answered, "I had better take black, as it's for a funeral."

Dr. Eccles thinks that a hot bath taken just before going to bed is valuable in producing deep, dreamless sleep, which, however, rarely lasts more than four hours. But four hours of good sleep would be a boon to many sufferers from wakeful nights.

The obstinate microbe will flourish in the most concentrated solution of carbolic acid, and is not to be killed by fairly-strong solutions of corrosive sublimate. In view of this, and of the fact that dangerous accidents often occur from carbolic acid, corrosive sublimate, and even iodoform, it is well that surgeons should recall the fact that plain water, filtered and boiled is one of the simplest and best of aseptics.

Dr. G. H. Fox is of opinion that alcohol, tea, coffee, and tobacco retard recovery in cases of skin disease, by interfering with the constancy of those processes of waste and repair which are necessary to the nutrition of the skin. Beer is the most injurious of all, and Dr. Fox says that in some cases he has thought the patient had better have taken a whole bottle of whisky than a single glass of malt liquor.

Dr. E. A. Wood, of Pittsburg, has been lecturing on racial developments, the three essential factors of which he holds to be heredity, leisure, and the kitchen. Beauty is to a great extent a matter of food, and he does not approve of children eating soft food which does not exercise the jaws. The leaders of mankind are the men with square jaws, the result of exercise and hard food. So far as our experience of children goes they have no passion for soft food, and will readily neglect their bread and milk if they can obtain a bone or a few nuts, or even a piece of slate pencil.

At the opening of a *creche* at Foggia, Dr. Recupito stated that in Italy more than three hundred thousand children die in infancy every year—a sufficient plea for the existence and foundation of that much-maligned charity the *creche.* He protested against the theory formulated by some economists (?) that it is legitimate to neglect children and the aged because they consume without producing, and a nation loses wealth and power when consumption exceeds production. Dr. Recupito says rightly that the old men form the brain of a nation, and the children its heart, and without heart and brain riches profit nothing and true power is impossible.

When you want to advertise do it thoroughly. A certain patent—we will not say quack—medicine is glorified in the *East London Advertiser,* under a really thrilling title—"A Man Without Blood: Remarkable Experiences of a London Hospital Patient." This individual suffered from a mysterious illness, which reduced him to such a condition that—at least so he says—he had not a drop of blood in his veins. Of course, the miraculous medicine he took to when the doctors despaired of him, restored his blood and everything else necessary to health and happiness; but, just as a matter of physiological curiosity, we should like to know how he defied the laws of nature by existing for an instant with "scarcely a drop of blood in his veins." He must be constituted on unique principles.

Madame Dumas, who laboured for many years among the women at the prison of St. Lazare, in Paris, was one who spared no trouble to help the unfortunate creatures to whom she devoted herself. When she was eighty-two years of age —she is now ninety-six—she set to work to learn Spanish, in order to comfort a young Andalusian girl who was in the prison, and who knew no other tongue.

Dr. T. R. Allinson has been trying the experiment of living on meal and water for a month. His daily allowance is one pound of whole meal made into a cake, with distilled water, and one quart of water. His account of his condition after a week is cheering. In the first few days he felt hungry, but about the fourth day this disappeared, and he had no longer any craving for other food. His brain was clear, his lung capacity had increased five inches, and both his sight and his hearing had improved. He had lost seven pounds weight, but seems to regard this as rather an advantage. Altogether he feels thoroughly satisfied with his experiment. It is a very economical one, the wheat for seven days having cost only eightpence. "This," he says, "is living on almost a penny a day and enjoying it."

We have already protested in these columns against substituting electricity for the gallows as a method of inflicting capital punishment. The change has been advocated on the ground of mercy, but on reading the account of the plan selected by the New York Medico-Legal Society after much deliberation as the method by which, after January 1st of this year, executions are to be carried out, one feels that it is hard to see where the mercy comes in. The criminal is bound to a table or fastened to a chair; one electrode should be arranged to impinge upon the spine between the shoulders, the other is to be attached to a sort of helmet that secures the head. The hair should be cut short, and the skin and poles of the electrodes made thoroughly wet with warm water. A powerful electric current should then be made to pass for thirty seconds, which will assuredly kill the criminal, and perhaps scorch him beyond recognition as well. Once begun the death is swift and sure enough, but as it is in the fear of death that its horror dwells, it may be questioned if these elaborate preparations for the fatal chair are really more merciful than the gallows and the drop.

One of the strongest proofs of the intelligence and sensitiveness of animals is shown in their extreme susceptibility to the influence of music. There is no doubt that the war-horse is inspired by the sound of military music; elephants have been trained to go through various evolutions to the sound of music from the earliest times, as may be seen by reference to the works of Pliny, Suetonius, and Plutarch; and the camel can be taught to walk in time to a warlike march. The emotion roused by music in dogs is well known, though their strong desire to join audibly in the concourse of sweet sounds is often misunderstood. But Buffon speaks of a dog which would leave its retreat in the kitchen at the sound of music, and return to its own domicile when the concert was over. We ourselves knew a dog who showed a similar taste, accompanied by a certain critical faculty. On hearing the piano he always made his way to the drawing-room; but if, when he arrived there, he found only scales and exercises going on, he would give vent to a sniff of indignation and walk downstairs again. The strains of Beethoven or Chopin, on the other hand, would have him up to the hearth-rug, where he would pass hours in a luxurious trance of bliss. If he had a preference in composers, it was for Beethoven, judging from the amount of attention he paid. Sterndale Bennett he only tolerated, and in one or two lengthy and complicated pieces seemed unable to distinguish between his work and Czerny's.

